# Complexity and Conservation of Thermospermine-Responsive uORFs of *SAC51* Family Genes in Angiosperms

**DOI:** 10.3389/fpls.2019.00564

**Published:** 2019-05-01

**Authors:** Soichi Ishitsuka, Mai Yamamoto, Minaho Miyamoto, Yoshitaka Kuwashiro, Akihiro Imai, Hiroyasu Motose, Taku Takahashi

**Affiliations:** Division of Earth, Life and Molecular Sciences, Graduate School of Natural Science and Technology, Okayama University, Okayama, Japan

**Keywords:** thermospermine, translational regulation, uORF, *SAC51*, ACL5, *Arabidopsis thaliana*

## Abstract

*ACAULIS5* (*ACL5*) encodes thermospermine synthase in Arabidopsis and its loss-of-function mutant *acl5* shows excess xylem differentiation and severe dwarfism. *SAC51* encodes a basic helix-loop-helix (bHLH) protein and was identified from *sac51-d*, a dominant suppressor mutant of *acl5*, which restores the wild-type phenotype without thermospermine. The 5′ leader of the *SAC51* mRNA contains multiple upstream open-reading frames (uORFs) and *sac51-d* has a premature stop codon in the fourth uORF. This uORF is conserved among *SAC51* family genes in vascular plants. According to the GUS reporter assay, the *SAC51* promoter was not responsive to thermospermine but the *SAC51* 5′ leader fused to the constitutive 35S promoter enhanced the GUS activity in response to thermospermine. Disruption experiments of each start codon of the *SAC51* uORFs revealed that uORF4 and uORF6 whose start codon corresponds to the second methionine codon of uORF4 had an inhibitory effect on the main ORF translation while the other four uORFs rather had a stimulatory effect. The response of the 5′ leader to thermospermine was retained after disruption of each one of six start codons of these uORFs but abolished by mutating both uORF4 and uORF6 start codons, suggesting the importance of the C-terminal sequence shared by these uORFs in the action of thermospermine. We introduced GUS fusions with 5′ leaders of *SAC51* family genes from other angiosperm species into Arabidopsis and found that all 5′ leaders responsive to thermospermine, so far examined, contained these two conserved, and overlapping uORFs.

## Introduction

Thermospermine is a structural isomer of spermine and present in some bacteria and ubiquitously in plants (Minguet et al., 2008; Fuell et al., 2010; Takano et al., 2012). In *Arabidopsis* *thaliana*, the *ACAULIS5* (*ACL5*) gene encodes thermospermine synthase (Knott et al., 2007; Kakehi et al., 2008) and is expressed exclusively in differentiating xylem vessels (Clay and Nelson, 2005). Its loss-of-function mutants show over-proliferation of xylem vessels and severe dwarfism (Hanzawa et al., 1997). Expression of the *ACL5* gene is up-regulated by auxin but down-regulated by thermospermine while the excess xylem phenotype of the *acl5* mutant is enhanced by synthetic and persistent auxin analogs but suppressed by thermospermine, indicating that both thermospermine synthesis and auxin-induced xylem differentiation are under negative feedback control by thermospermine (Yoshimoto et al., 2012). Except in Arabidopsis, however, the function of thermospermine in xylem development has only been investigated in few species including poplar (Milhinhos et al., 2013) and cotton (Mo et al., 2015). On the other hand, we have developed an artificial inhibitor of thermospermine synthase, named xylemin, which showed a potent inducing effect of ectopic xylem formation in tobacco leaves in the presence of a synthetic auxin analog (Yoshimoto et al., 2016). Thus, in view of biotechnological applications, the combinatorial use of these plant growth regulators might have a potential for the control of woody biomass.

To clarify the mode of action of thermospermine, previous studies have identified suppressor mutants named *suppressor of acl5* (*sac*) that rescue the *acl5* phenotype without thermospermine. The results revealed that *SAC51* encodes a basic helix-loop-helix (bHLH) transcription factor (Imai et al., 2006) while *SAC52*, *SAC53*, and *SAC56* encode ribosomal proteins, RPL10, RACK1, and RPL4, respectively (Imai et al., 2008; Kakehi et al., 2015). *SAC51* contains six upstream open reading frames (uORFs) in the 5′ leader region of the mRNA. A dominant allele, s*ac51-d*, has a premature stop codon in the fourth uORF. uORFs are highly abundant in the genomes of angiosperms and the fourth uORF of *SAC51* is one of the highly conserved uORFs among different plant species (Hayden and Jorgensen, 2007). Conserved uORFs are generally present in regulatory genes and have an inhibitory effect on the main ORF translation, which is often attenuated by ribosomal translation reinitiation (Tran et al., 2008; Jorgensen and Dorantes-Acosta, 2012; von Arnim et al., 2014). Thermospermine may specifically counteract this effect on the *SAC51* mRNA and lead to translation of the main ORF. Dominant or semi-dominant alleles of *SAC52*, *SAC53*, and *SAC56*, have been suggested to enhance translation of the *SAC51* main ORF instead of thermospermine (Kakehi et al., 2015). *SAC51* in turn may be involved in repressing the expression of *ACL5* and a subset of genes required for xylem differentiation. Another study identified point mutations of the conserved uORF in *SACL1* and *SACL3* of the same *SAC51* family as suppressors of *acl5* (Vera-Sirera et al., 2015). We also found that *sac57-d* has a point mutation in this conserved uORF of *SACL3* (Cai et al., 2016).

upstream open-reading frames-dependent translation regulated by polyamines is well known for S-adenosylmethionine decarboxylase (AdoMetDC). Mammalian *AdoMetDC* mRNAs contain a conserved uORF encoding the hexapeptide MAGDIS (Ruan et al., 1996). Ribosomes synthesizing this peptide are stalled by high concentrations of polyamines and blocked to access to the main ORF encoding the enzyme that catalyzes the production of decarboxylated AdoMet, an aminopropyl group donor for the synthesis of polyamines. In Arabidopsis, the *AdoMetDC1* mRNA contains two conserved uORFs that are overlapping in different frames and have also been shown to regulate the translation of the main ORF according to polyamine levels (Franceschetti et al., 2001; Hanfrey et al., 2005). In mammals and yeasts, high levels of polyamines cause +1 and −2 ribosomal frameshifting, respectively, during the translation of the ornithine decarboxylase (ODC) antizyme mRNA, which contains an extra nucleotide in the coding sequence, and lead to the synthesis of a functional protein that mediates degradation of ODC, a rate-limiting enzyme in polyamine biosynthesis (Matsufuji et al., 1996). This regulatory mechanism may not be conserved in plants (Ivanov and Atkins, 2007). On the other hand, the translational enhancement by thermospermine has been reported so far only for Arabidopsis *SAC51* family members (Takahashi, 2018). Our studies have shown that, among the four members of the *SAC51* family, *SAC51* and *SACL1* are responsive to thermospermine but *SACL2*, and *SACL3* are not (Cai et al., 2016; Yamamoto and Takahashi, 2017). We were thus interested in determining how much the response mechanism to thermospermine is conserved across plant species. Here we carried out a more detailed study of the responsiveness to thermospermine of the 5′ leader region of the *SAC51* mRNA and also extended our analysis to that of *SAC51* family genes in different angiosperm species.

## Materials and Methods

### Plant Material and Growth Conditions

*Arabidopsis thaliana* Col-0 accession was used as the wild-type plant. Seeds were surface-sterilized in bleach solution containing 0.01% (v/v) Triton X-100 for 3 min, washed 3 times with sterile water, and sown onto MS medium (Murashige and Skoog, 1962) containing 3% sucrose and 0.8% agar. Plants were grown under 16 h light and 8 h dark at 22°C. For thermospermine treatment, seedlings were incubated for 24 h in liquid MS medium with 100 μM thermospermine-4HCl, which was purchased from Santa Cruz Biotechnology.

### Genomic DNA Preparation

Arabidopsis genomic DNA was prepared as described previously (Imai et al., 2006). Genomic DNAs of broccoli (*Brassica oleracea*), soybean (*Glycine max*), poplar (*Populus tremula × alba*), and rice (*Oryza sativa*) were prepared from each seedling by using NucleoSpin Plant II kit (Macherey-Nagel) following the manufacturer’s instruction.

### T-DNA Construction and Plant Transformation

For the *SAC51* promoter-driven expression of the GUS reporter gene, a 990-bp genomic fragment upstream from the first exon of *SAC51* was amplified by PCR with primers, SAC51-proFCl, and SAC51-proRBg (Supplementary Table S1), cloned into a pGEM-T easy vector (Promega), and then transferred as a *Cla*I-*Bgl*II fragment to *Cla*I-*Baml*HI sites of Ti-plasmid vector pBI101 (Clontech). The GUS gene construct fused to the *SAC51* promoter with the first untranslated exon and intron was similarly made using primers, SAC51-proFCl, and SAC51-ex2RBg (Supplementary Table S1). The GUS construct containing the *SAC51* promoter and the whole 5′ leader region has been described previously (Imai et al., 2006). The mutant versions were generated by PCR-based site-directed mutagenesis as follows. First-round PCR was performed in 10 cycles of two separate reactions using the wild-type construct as a template with a primer pair of SAC51-proFCl and SAC51-mXR or with a primer pair of SAC51-mXF and SAC51-5RBg (Supplementary Table S1). The PCR products were mixed and subsequently amplified by 10 cycles of PCR with a primer pair of SAC51-proFCl and SAC51-5RBg. The product from a second round of PCR was cloned into pGEM-T Easy and transferred as a *Cla*I-*Bgl*II fragment to pBI101. The no-uORF version was generated sequentially by introducing a point mutation in the start codon of each uORF.

To generate the CaMV 35S promoter-GUS construct containing a genomic or cDNA fragment of the 5′ leader region of *SAC51*, the fragment was amplified from genomic DNA or reverse-transcribed cDNA by PCR with primers, SAC51-5FSp and SAC51-5RBg (Supplementary Table S1), cloned into pGEM-T easy, and then inserted as a *Spe*I- *Bgl*II fragment between the 35S promoter and the GUS coding region of pBI121 (Clontech). Point mutations were sequentially introduced into the start codon of each uORF as described above. The other 35S-5′-GUS constructs containing a 5′ leader region of SAC51 homologs from different plant species were made in a similar way. All PCR was performed using Ex Taq DNA polymerase (Takara) according to the manufacturers protocol. PCR conditions were 50 cycles of 94°C for 30 s, 55°C for 30 s, and 72°C for 90 s, unless otherwise stated. The primers used were shown in Supplementary Table S1.

Ti plasmid constructs were introduced into *Agrobacterium tumefaciens* C58C1 by electroporation (Mersereau et al., 1990). Arabidopsis plants were transformed using the floral dip method (Clough and Bent, 1998). Transgenic lines were selected on kanamycin and confirmed by PCR using PBI-Cl and GUS primers (Supplementary Table S1). At least five independent homozygous lines carrying single copy of the transgene were further selected based on the segregation ratio of kanamycin-resistant plants in subsequent generations.

### GUS Assays

Fluorometric assay of GUS activity was performed as described previously (Jefferson et al., 1987). The fluorescence was measured with an RF-5300PC spectrofluorophotometer (Shimadzu, Japan). Total protein content was measured by using the Bradford assay (BioRad). For histochemical staining of GUS activity, samples were prefixed for 20 min in ice-cold 90% (v/v) acetone under vacuum, rinsed three times with water, and incubated in GUS staining buffer containing 50 mM Na_2_HPO_4_/NaH_2_PO_4_ (pH7.0), 2 mM K_3_Fe(CN)_6_, 2 mM K_4_Fe(CN)_6_, 0.1% Triton-X100, and 1 mM 5-bromo-4-chloro-3-indolyl glucuronide, at 37°C overnight. Samples were then treated with 70% ethanol to remove chlorophyll.

### Quantitative RT-PCR Analysis

Total RNA was extracted from Arabidopsis seedlings by the SDS-phenol method (Hanzawa et al., 1997) and reverse transcribed by using the PrimeScript II first strand cDNA Synthesis Kit (Takara). Quantitative RT-PCR was performed on the Thermal Cycler Dice TP760 (Takara) using the KAPA SYBR FAST Universal (KAPA Biosystems). *ACTIN8* (*ACT8*) was used as a reference gene for normalization. Means of expression levels were calculated from three technical replicates. Primers used were ACT8-F (5′-GTGAG CCAGA TCTTC ATTCG TC-3′) and ACT8-R (5′-TCTCT TGCTC GTAGT CGACA G-3′) for ACT8 and SAC51-FF (5′-CATTC CTTTC TAAGA TACTA AAG-3′) and GUS (Supplementary Table S1) for the *SAC51* 5′-fused *GUS*, respectively.

### *In vitro* Transcription and Translation

The GFP reporter gene was amplified by PCR from pEGFP (Clontech) using primers, GFP-ATG and GFP-3 (Supplementary Table S1), and cloned as a *Bam*HI-*Xho*I fragment into pT7 Blue-2 (Promega) to generate a control plasmid, pSI020. For the *SAC51* 5′ leader-GFP transcriptional fusion construct, an 860-bp cDNA fragment of the *SAC51* 5′ leader region was amplified by PCR with primers, SAC51-5FBal, and SAC51-5RBal (Supplementary Table S1), and inserted into the *Bal*I restriction site of pSI020 just upstream of the GFP coding sequence. The no-uORF version of the *SAC51* 5′ leader was generated by PCR from that of the 35S-*SAC51* 5′-GUS T-DNA and similarly cloned into pSI020.

These plasmids were digested with *Xba*I to generate linear DNA templates for transcription and transcribed using T7 RNA polymerase in the presence of Ribo m7G cap analog (Promega). The resulting capped RNAs were translated in wheat germ extract with Transcend biotinylated lysine-tRNA (Promega) in the presence or absence of thermospermine according to the manufacturer’s instructions.

*In vitro*-translated proteins were subjected to SDS–polyacrylamide gel electrophoresis, transferred to PVDF membrane, and detected using Transcend non-radioactive translation detection systems (Promega). The gel images were visualized using a LAS-4000 mini luminescent imaging analyzer (Fujifilm).

### Statistical Analysis

All statistical analyses were performed using the EZR software (Kanda, 2013), which is a graphical user interface for R (The R Foundation for Statistical Computing). Significance of differences between groups was estimated by one-way or two-way ANOVA with Tukey-Kramer multiple comparisons test.

## Results

### The *SAC51* Promoter Is Not Responsive to Thermospermine

**FIGURE 1:**
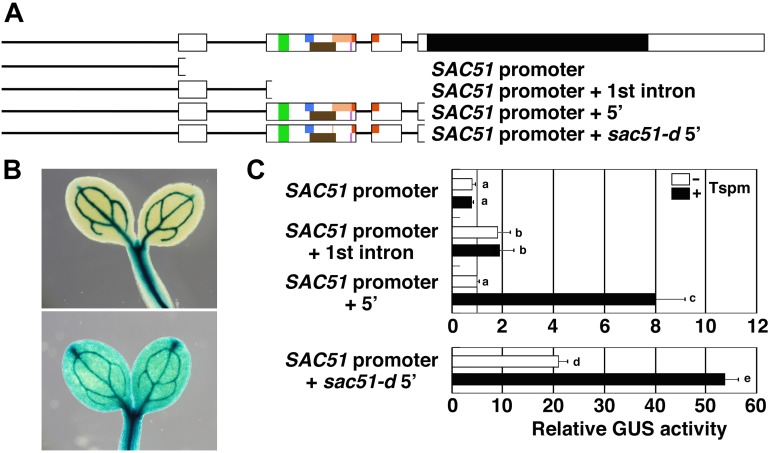
The response of the *SAC51* promoter and 5’ leader regions to thermospermine. **(A)** Gene structure of *SAC51* and the regions used for GUS fusion constructs. Bars are promoter regions and introns of *SAC51*. Exons are shown in white boxes in which colored and black areas represent uORFs and a main ORF, respectively. *sac51-d* contains a premature stop codon in uORF4. **(B)** GUS staining patterns in cotyledons carrying the GUS fusion with only the *SAC51* promoter (upper panel) and that with the *SAC51* promoter-5′ leader (lower panel). **(C)** Relative GUS activity of GUS fusion constructs. Ten-day-old seedlings were treated with or without 100 μM thermospermine for 24 h before GUS assays. Results are from single representative transgenic lines for each construct and the GUS activity of the *SAC51* promoter-5′ leader-GUS fusion without thermospermine is set as 1. Error bars represent SD (*n* = 4). Different letters indicate statistically significant differences between means by two-way ANOVA with Tukey–Kramer multiple comparison test (*P* < 0.05).

There are six uORFs in the 5′ leader region of the *SAC51* mRNA, including the sixth uORF, which presumptively starts from an in-frame start codon, namely, the second methionine codon of the fourth uORF (Yamamoto and Takahashi, 2017). These uORFs are encoded in the second and third exons of *SAC51*. To confirm whether more upstream regions are responsive to thermospermine or not, we generated transgenic lines carrying the GUS reporter gene under the control of the *SAC51* promoter or that followed by the first exon and intron with its splice acceptor site (Figure 1A) and examined the GUS activity. The results showed that the *SAC51* promoter directed the GUS expression sharply to vascular cells (Figure 1B) and neither only the promoter nor the promoter followed by the first exon and intron was responsive to thermospermine (Figure 1C). However, when the *SAC51* upstream regions containing the whole 5′ leader sequence was used, weak GUS expression was detected in additional tissues to the vasculature (Figure 1B) and the GUS activity was increased by 24-h treatment with thermospermine (Figure 1C), as described previously (Kakehi et al., 2008). The *sac51-d* mutant construct in which the fourth uORF contains a premature stop codon (Figure 1A) shows much higher GUS expression than the wild-type construct (Imai et al., 2006) but still retained the responsiveness to thermospermine (Figure 1C).

### Both uORF4 and uORF6 of *SAC51* Are Responsive to Thermospermine

**FIGURE 2:**
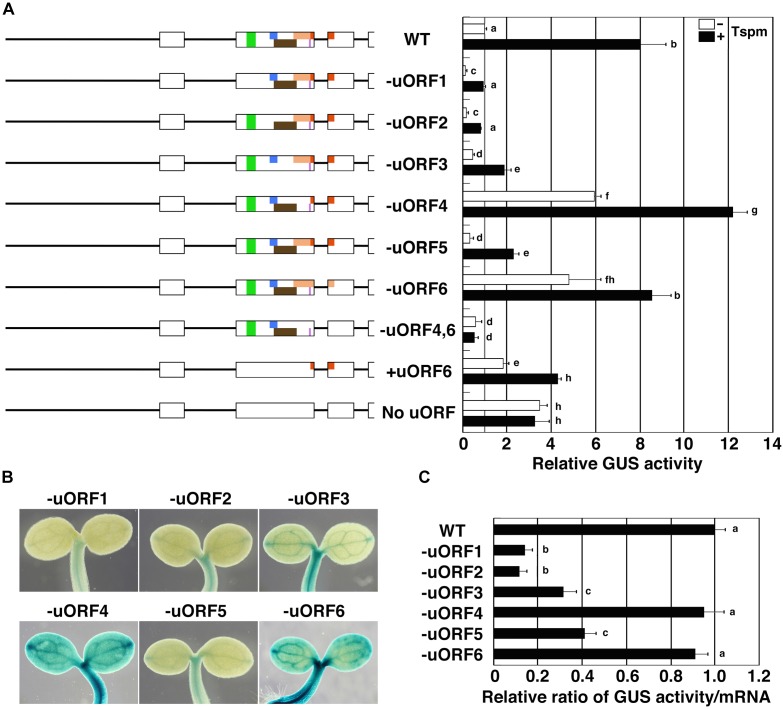
Effect of disruption of each start codon of *SAC51* uORFs on the GUS activity. **(A)** The regions used for GUS fusion constructs and relative GUS activity of each construct. Assays were performed as shown in Figure 1C. Error bars represent SD (*n* = 4). Different letters indicate statistically significant differences between means by two-way ANOVA with Tukey–Kramer multiple comparison test (*P* < 0.05). **(B)** GUS staining patterns in cotyledons carrying the GUS fusion with the *SAC51* promoter-5′ leader of each mutant uORF version. **(C)** Relative ratio of the GUS activity to its mRNA level. The GUS activity and the *GUS* mRNA level were measured using 10-day-old seedlings of each transgenic line. Error bars represent SD (*n* = 4). Different letters indicate statistically significant differences between means by one-way ANOVA with Tukey–Kramer multiple comparison test (*P* < 0.05).

To address which uORFs of the *SAC51* mRNA are involved in the response to thermospermine, we generated transgenic lines that carry the GUS fusion constructs with the *SAC51* promoter and 5′ leader containing a base substitution in the start codon of each uORF (Figure 2A) and examined the effect of thermospermine on the GUS activity. All constructs in which one of the uORF start codons is mutated were shown to retain the response to thermospermine. As also shown by GUS staining (Figure 2B), the results revealed that the basal GUS activity was rather reduced in uORF1, uORF2, uORF3, and uORF5-disruption constructs and increased in uORF4 and uORF6-disruption constructs compared with that in the wild-type construct, suggesting that uORF4 and uORF6 are inhibitory but uORF1, uORF2, uORF3, and uORF5 are stimulatory to the main ORF translation. We confirmed by RT-PCR experiments that the relative ratio of the GUS activity to the GUS transcript level was reduced in transgenic lines with uORF1, uORF2, uORF3, and uORF5-disruption constructs (Figure 2C). Disruption of uORF4 or uORF6 appeared to reduce the responsiveness to thermospermine. Our previous study suggests the importance of this uORF6 because the 5′ leaders of both *SAC51* and *SACL1* contain this in-frame uORF and are responsive to thermospermine but those of *SACL2* and *SACL3* don’t contain it and show no response to thermospermine (Yamamoto and Takahashi, 2017). We thus disrupted start codons of both uORF4 and uORF6 and found that this mutant construct resulted in no response to thermospermine (Figure 2A). Disruption of all of six start codons of the uORFs also caused no response to thermospermine but the construct with the loss of all but the uORF6 start codon showed the response (Figure 2A). These results collectively suggest the requirement for at least one of uORF4 and uORF6 in the response to thermospermine.

**FIGURE 3:**
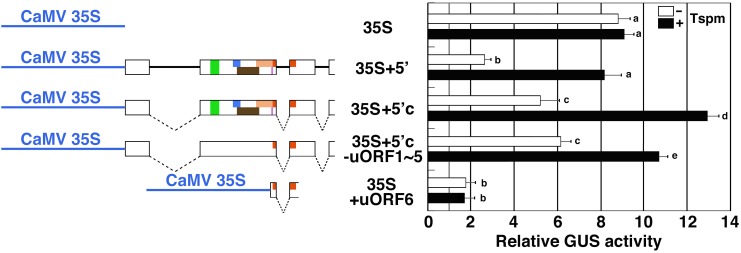
The response of the *SAC51* 5′ leader region fused with the CaMV 35S promoter to thermospermine. The regions used for GUS fusion constructs and relative GUS activity of GUS fusion constructs are shown. Dashed lines indicate introns spliced out in the cDNA constrcuts. Assays were performed as shown in Figure 1C. Error bars represent SD (*n* = 4). Different letters indicate statistically significant differences between means by two-way ANOVA with Tukey–Kramer multiple comparison test (*P* < 0.05).

We also examined the response of the *SAC51* 5′ leader region to thermospermine under the control of the constitutive cauliflower mosaic virus (CaMV) 35S promoter. Although the 35S promoter is not responsive to thermospermine, insertion of a genomic or cDNA fragment of the *SAC51* 5′ leader region between the promoter and the GUS reporter gene conferred the response to thermospermine in the GUS activity (Figure 3). Furthermore, the mutant cDNA construct in which all but uORF6 is disrupted was responsive to thermospermine whereas the 5′ fragment containing the minimum uORF6 alone resulted in no response (Figure 3).

### The Response to Thermospermine Is Conserved in Dicots and Monocots

**FIGURE 4:**
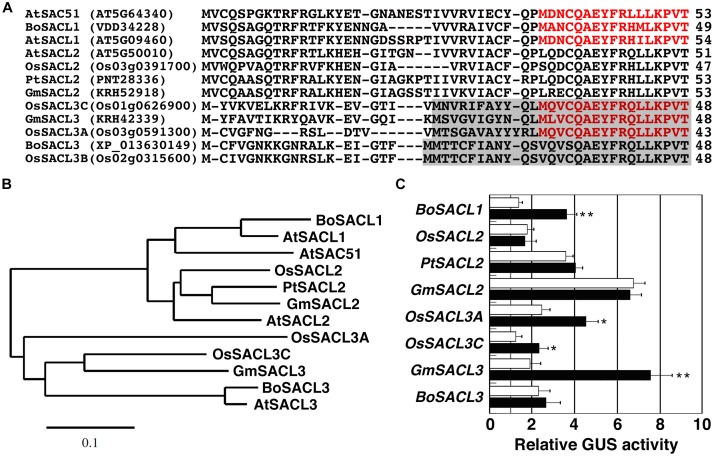
Comparison of the conserved uORFs of *SAC51* family genes. **(A)** Alignment of amino acid sequences deduced from the conserved uORFs of *SAC51* family genes from different plant species. The amino acids encoded by the internal uORF corresponding to uORF6 of *AtSAC51* are colored in red and those encoded by another uORF in SACL3 homologs are shaded. Dashes indicate gaps. *At*, *Arabidopsis thaliana*; *Bo*, *Brassica oleracea*; *Os*, *Oryza sativa*; *Pt*, *Populus trichocarpa*; *Gm*, *Glycine max*. Gene ID or GenBank accession numbers are given in parentheses. **(B)** Phylogenetic relationship of the conserved uORFs shown in **(A)**. The tree based on the deduced amino acid sequences was constructed using the neighbor-joining method of the MEGA7 software (Kumar et al., 2016). The scale bar indicates the number of amino acid substitutions per site. **(C)** The response of each 5′ leader region fused with the CaMV 35S promoter to thermospermine. GUS assays were performed as shown in Figure 1C. Error bars represent SD (*n* = 4). Asterisks indicate a significant increase as compared with control (*t*-test, ^∗^*P* < 0.05, ^∗∗^*P* < 0.01).

Because the uORF4 of *SAC51* is widely conserved in *SAC51* family genes of different plant species, we examined whether 5′ leader regions of these mRNAs are responsive to thermospermine or not. The 5′ regions were cloned from broccoli, soybean, poplar, and rice genomic DNA, inserted between the 35S promoter and the GUS gene, and introduced into Arabidopsis. The deduced amino acid sequences of the conserved uORFs, some of which contain in-frame ATG codons, namely, in-frame uORFs, are aligned in Figure 4A, and their phylogenetic relationships are shown in Figure 4B. We detected the response to thermospermine in some constructs including *BoSACL1*, *OsSACL3A*, *OsSACL3C*, and *GmSACL3*, but not in others including *OsSACL2*, *PtSACL2*, *GmSACL2*, and *BoSACL3* (Figure 4C).

### The Inhibitory Effect of the 5′ Leader on Translation Is Repressed *in vitro* by Heat

**FIGURE 5:**
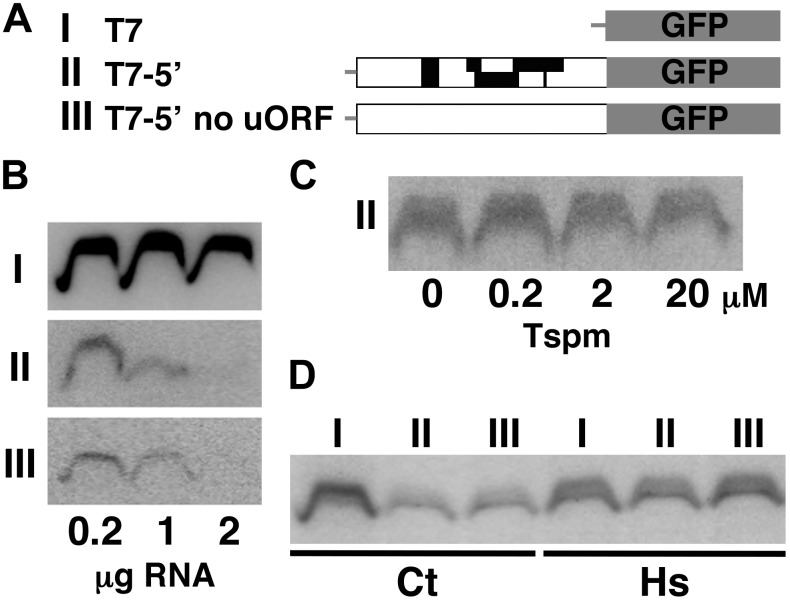
*In vitro* translation of the *SAC51* 5′ leader-*GFP* fusion transcript using a wheat-germ extract. **(A)** Structure of the three constructs used. The constructs of *GFP* alone (I), *GFP* fused with the *SAC51* 5′ leader (II), and *GFP* fused with the *SAC51* 5′ leader in which all start codons of uORFs are mutated (III), were transcribed *in vitro* using T7 RNA polymerase. **(B)** Effect of the amount of substrate transcripts on GFP synthesis. **(C)** Effect of thermospermine on GFP synthesis. 0.4 μg each of the *in vitro* transcript from the construct II was used in the translation reaction. **(D)** Effect of pretreatment by heat of the transcripts on GFP synthesis. 0.4 μg each of the in-vitro transcripts was treated at 25°C (Ct) or 65°C (Hs) for 10 min before translation.

We finally tested whether or not the response of the *SAC51* transcript to thermospermine can be reproduced *in vitro*. The cDNA fragment of the *SAC51* 5′ leader region was fused to the GFP reporter gene (Figure 5A), transcribed *in vitro*, and then translated by using a wheat germ extract translation system. Detection of the chemiluminescent-labeled GFP protein showed that the efficiency of GFP translation was reduced by adding the *SAC51* 5′ leader sequence of both wild-type and no-uORF versions to the GFP transcript and further reduced by increasing the amount of the transcript in the translation reaction (Figure 5B). Addition of thermospermine to the translation reaction mixture had no effect on the GFP production (Figure 5C). We found, however, that pretreatment of the 5′-GFP fusion transcript of both wild-type and no-uORF versions with heat at 65°C for 10 min effectively increased the translation efficiency (Figure 5D).

## Discussion

The results of GUS expression experiments, first of all, confirmed that the *SAC51* promoter, the first exon, which contains no coding sequence, and the first intron are not responsive to thermospermine. On the other hand, the full-length 5′ leader region solely conferred the responsiveness to thermospermine on its fused mRNA under a certain promoter. We have previously shown that the mRNA levels of *SAC51* and *SACL1* are increased by 24-h treatment with thermospermine (Kakehi et al., 2010; Cai et al., 2016). Because these mRNAs have also been identified as non-sense-mediated mRNA decay (NMD) targets (Kurihara et al., 2009), these increases may be caused by enhancement of the main ORF translation followed by the avoidance of NMD. We thus conclude that the response of *SAC51* to thermospermine is predominantly regulated at the level of translation. A previous study of the *SAC51* expression in root tissue has shown that it is broadly expressed within the vascular cylinder (Vera-Sirera et al., 2015). *ACL5* expression is more restricted to differentiating xylem vessels (Katayama et al., 2015). Given that thermospermine is a mobile signal, its concentration gradient could potentially control spatiotemporal distribution of the SAC51 protein through enhancing its mRNA translation. The mechanism of cell-to-cell transport of thermospermine remains, and needs to be addressed.

Disruption of the conserved uORF4 or its internal uORF6 increased basal levels of the GUS activity, suggesting their inhibitory role in the main ORF translation. In contrast, disruption of each single uORF of uORF1, uORF2, uORF3, and uORF5, which are not conserved among plant species, rather reduced basal levels of the GUS reporter activity. It is possible that these uORFs serve to lead the scanning ribosomes to bypass the conserved uORF4 and its internal uORF6 and reinitiate translation from the downstream main ORF to some extent. Disruption of both uORF4 and uORF6 completely abolished the response to thermospermine. Thus, these two uORFs may play a cooperative or redundant role in the regulation of the *SAC51* main ORF translation, although at least uORF6 alone within the context of the full-length 5′ leader region is sufficient for the response to thermospermine. Simultaneous disruption of these uORFs also reduced the basal level of the GUS activity. It is possible that point mutations in these uORFs alter the secondary structure of the 5′ leader region of the transcript, thereby affecting the translation efficiency of the main ORF. The internal uORF6 within uORF4 is also present in *SACL1* but not in *SACL2* and *SACL3*. We have previously suggested the importance of this short uORF because *SACL2* and *SACL3* show no clear response to thermospermine (Yamamoto and Takahashi, 2017). However, it should be noted that the response to thermospermine was still detected in the case of the uORF6 disruption construct. More detailed studies such as the construction of synonymous substitutions of the conserved uORF6 will be necessary.

The response of *SAC51* family genes to thermospermine has not been investigated before in other plant species than Arabidopsis. Our results showed that 5′ leader regions of *Brassica SACL1*, soybean *SACL3*, rice *SACL3A*, and *SACL3C*, were responsive to thermospermine in transgenic Arabidopsis plants. All of these contain at least the two uORFs corresponding to the conserved uORF4 and uORF6 of the Arabidopsis *SAC51*, suggesting again the relevance of these two uORFs to the response to thermospermine. Although *SACL3*-homologous mRNAs tested contain another uORF in the conserved uORF (Figure 4A), it may not always relate to the response to thermospermine. Further analysis of *SACL3*-homologous mRNAs in different plant species will give a clue as to the arrangement of uORFs responsive to thermospermine. In Arabidopsis, TMO5-LHW and T5L1-LHW heterodimeric transcription factors have been shown to commonly regulate expression of *ACL5* and *SACL3* in xylem precursor cells in the root (Katayama et al., 2015). Thus, translational response to thermospermine might no longer be critical for *SACL3* expression. On the other hand, *SACL2*-homologous mRNAs tested contain no additional uORF in the conserved uORF and showed no response to thermospermine. These results pose a question about the significance of the conserved uORF in the response to thermospermine.

Unfortunately, the response of the 5′ leader region of *SAC51* to thermospermine was not reproduced in the wheat germ *in vitro* translation system. It is possible that additional mediators are required for the function of thermospermine as is the case for other plant hormone signals, which are mediated by hormone receptor proteins. However, given the mode of action of polyamines in mRNA translation so far identified and their strong affinity to nucleic acids (Igarashi and Kashiwagi, 2015), it is most likely that thermospermine directly interacts with RNA molecules. Thermospermine might be required to integrate with ribosomal RNA during the formation of small and large ribosomal subunits although the possibility cannot be excluded that thermospermine interacts with a specific mRNA sequence such as the *SAC51* 5′ leader region and alters its secondary structure to work as a probable thermospermine-responsive riboswitch. The *in vitro* transcribed *SAC51* 5′ leader was not responsive to thermospermine but to heat treatment, suggesting the importance of the secondary structure of the 5′ leader sequence in translation. Whether or not all the uORFs are intact, the long 5′ leader of *SAC51* reduced GFP translation as the amount of the transcript added to the reaction increased, suggesting the need for more ribosomes in the reaction. Further investigation of optimal reaction conditions may be required. It would also be interesting and worth to examine whether or not the translational enhancement by thermospermine can be reproduced and applied as a biotechnological tool in animal and fungal systems.

## Author Contributions

SI, YK, AI, HM, and TT designed the experiments. SI, MY, MM, and YK carried out the experiments and analyzed the data. SI and TT wrote the manuscript.

## Conflict of Interest Statement

The authors declare that the research was conducted in the absence of any commercial or financial relationships that could be construed as a potential conflict of interest.

## References

[B1] Cai Q. Fukushima H. Yamamoto M. Ishii N. Sakamoto T. Kurata T. (2016). The SAC51 family plays a central role in thermospermine responses in Arabidopsis. *Plant Cell Physiol.* 57 1583–1592. 10.1093/pcp/pcw113 27388339

[B2] Clay N. K. Nelson T. (2005). Arabidopsis *thickvein* mutation affects vein thickness and organ vascularization, and resides in a provascular cell-specific spermine synthase involved in vein definition and in polar auxin transport. *Plant Physiol.* 138 767–777. 10.1104/pp.104.055756 15894745 PMC1150395

[B3] Clough S. J. Bent A. F. (1998). Floral dip: a simplified method for Agrobacterium-mediated transformation of *Arabidopsis thaliana*. *Plant J.* 16 735–743. 10.1046/j.1365-313x.1998.00343.x 10069079

[B4] Franceschetti M. Hanfrey C. Scaramagli S. Torrigiani P. Bagni N. Burtin D. (2001). Characterization of monocot and dicot plant S-adenosyl-l-methionine decarboxylase gene families including identification in the mRNA of a highly conserved pair of upstream overlapping open reading frames. *Biochem. J.* 353 403–409. 10.1042/bj3530403 11139406 PMC1221584

[B5] Fuell C. Elliott K. A. Hanfrey C. C. Franceschetti M. Michael A. J. (2010). Polyamine biosynthetic diversity in plants and algae. *Plant Physiol. Biochem.* 48 513–520. 10.1016/j.plaphy.2010.02.008 20227886

[B6] Hanfrey C. Elliott K. A. Franceschetti M. Mayer M. J. Illingworth C. Michael A. J. (2005). A dual upstream open reading frame-based autoregulatory circuit controlling polyamine-responsive translation. *J. Biol. Chem.* 280 39229–39237. 10.1074/jbc.m509340200 16176926

[B7] Hanzawa Y. Takahashi T. Komeda Y. (1997). ACL5: an *Arabidopsis* gene required for internodal elongation after flowering. *Plant J.* 12 863–874. 10.1046/j.1365-313x.1997.12040863.x 9375398

[B8] Hayden C. A. Jorgensen R. A. (2007). Identification of novel conserved peptide uORF homology groups in *Arabidopsis* and rice reveals ancient eukaryotic origin of select groups and preferential association with transcription factor-encoding genes. *BMC Biol.* 5:32. 10.1186/1741-7007-5-32 17663791 PMC2075485

[B9] Igarashi K. Kashiwagi K. (2015). Modulation of protein synthesis by polyamines. *IUBMB Life* 67 160–169. 10.1002/iub.1363 25906835

[B10] Imai A. Hanzawa Y. Komura M. Yamamoto K.-T. Komeda Y. Takahashi T. (2006). The dwarf phenotype of the *Arabidopsis* acl5-1 mutant is suppressed by a mutation in an upstream ORF of a bHLH gene. *Development* 133 3575–3585. 10.1242/dev.02535 16936072

[B11] Imai A. Komura M. Kawano E. Kuwashiro Y. Takahashi T. (2008). A semi-dominant mutation in the ribosomal protein L10 gene suppresses the dwarf phenotype of the acl5 mutant in Arabidopsis. *Plant J.* 56 881–890. 10.1111/j.1365-313X.2008.03647.x 18694459

[B12] Ivanov I. P. Atkins J. F. (2007). Ribosomal frameshifting in decoding antizyme mRNAs from yeast and protists to humans: close to 300 cases reveal remarkable diversity despite underlying conservation. *Nucleic Acids Res.* 35 1842–1858. 10.1093/nar/gkm035 17332016 PMC1874602

[B13] Jefferson R. A. Kavanagh T. A. Bevan M. W. (1987). GUS fusions: beta-glucuronidase as a sensitive and versatile gene fusion marker in higher plants. *EMBO J.* 6 3901–3907. 10.1002/j.1460-2075.1987.tb02730.x 3327686 PMC553867

[B14] Jorgensen R. A. Dorantes-Acosta A. E. (2012). Conserved peptide upstream open reading frames are associated with regulatory genes in angiosperms. *Front. Plant Sci.* 3:191. 10.3389/fpls.2012.00191 22936940 PMC3426882

[B15] Kakehi J.-I. Kawano E. Yoshimoto K. Cai Q. Imai A. Takahashi T. (2015). Mutations in ribosomal proteins, RPL4 and RACK1, suppress the phenotype of a thermospermine-deficient mutant of *Arabidopsis thaliana*. *PLoS One* 27:e0117309. 10.1371/journal.pone.0117309 25625317 PMC4308196

[B16] Kakehi J.-I. Kuwashiro Y. Motose H. Igarashi K. Takahashi T. (2010). Norspermine substitutes for thermospermine in the control of stem elongation in *Arabidopsis thaliana*. *FEBS Lett.* 584 3042–3046. 10.1016/j.febslet.2010.05.035 20580714

[B17] Kakehi J.-I. Kuwashiro Y. Niitsu M. Takahashi T. (2008). Thermospermine is required for stem elongation in *Arabidopsis thaliana*. *Plant Cell Physiol.* 49 1342–1349. 10.1093/pcp/pcn109 18669523

[B18] Kanda Y. (2013). Investigation of the freely available easy-to-use software ‘EZR’ for medical statistics. *Bone Marrow Transplant.* 48 452–458. 10.1038/bmt.2012.244 23208313 PMC3590441

[B19] Katayama H. Iwamoto K. Kariya Y. Asakawa T. Kan T. Fukuda H. (2015). A negative feedback loop controlling bHLH complexes is involved in vascular cell division and differentiation in the root apical meristem. *Curr. Biol.* 25 3144–3150. 10.1016/j.cub.2015.10.051 26616019

[B20] Knott J. M. Römer P. Sumper M. (2007). Putative spermine synthases from *Thalassiosira pseudonana* and Arabidopsis thaliana synthesize thermospermine rather than spermine. *FEBS Lett.* 581 3081–3086. 10.1016/j.febslet.2007.05.074 17560575

[B21] Kumar S. Stecher G. Tamura K. (2016). MEGA7: molecular evolutionary genetics analysis version 7.0 for bigger datasets. *Mol. Biol. Evol.* 33 1870–1874. 10.1093/molbev/msw054 27004904 PMC8210823

[B22] Kurihara Y. Matsui A. Hanada K. Kawashima M. Ishida J. Morosawa T. (2009). Genome-wide suppression of aberrant mRNA-like noncoding RNAs by NMD in *Arabidopsis*. *Proc. Natl. Acad. Sci. U.S.A.* 106 2453–2458. 10.1073/pnas.0808902106 19181858 PMC2650177

[B23] Matsufuji S. Matsufuji T. Wills N. M. Gesteland R. F. Atkins J. F. (1996). Reading two bases twice: mammalian antizyme frameshifting in yeast. *EMBO J.* 15 1360–1370. 10.1002/j.1460-2075.1996.tb00478.x 8635469 PMC450040

[B24] Mersereau M. Pazour G. J. Das A. (1990). Efficient transformation of *Agrobacterium tumefaciens* by electroporation. *Gene* 90 149–151. 10.1016/0378-1119(90)90452-w 2165971

[B25] Milhinhos A. Prestele J. Bollhöner B. Matos A. Vera-Sirera F. Rambla J. L. (2013). Thermospermine levels are controlled by an auxin-dependent feedback loop mechanism in *Populus* xylem. *Plant J.* 75 685–698. 10.1111/tpj.12231 23647338

[B26] Minguet E. G. Vera-Sirera F. Marina A. Carbonell J. Blázquez M. A. (2008). Evolutionary diversification in polyamine biosynthesis. *Mol. Biol. Evol.* 25 2119–2128. 10.1093/molbev/msn161 18653732

[B27] Mo H. Wang X. Zhang Y. Yang J. Ma Z. (2015). Cotton ACAULIS5 is involved in stem elongation and the plant defense response to *Verticillium dahliae* through thermospermine alteration. *Plant Cell Rep.* 34 1975–1985. 10.1007/s00299-015-1844-3 26209974

[B28] Murashige T. Skoog F. (1962). A revised medium for rapid growth and bioassays with tobacco tissue cultures. *Physiol. Plant.* 15 473–497. 10.1111/j.1399-3054.1962.tb08052.x

[B29] Ruan H. Shantz L. M. Pegg A. E. Morris D. R. (1996). The upstream open reading frame of the mRNA encoding S-adenosylmethionine decarboxylase is a polyamine-responsive translational control element. *J. Biol. Chem.* 271 29576–29582. 10.1074/jbc.271.47.29576 8939886

[B30] Takahashi T. (2018). Thermospermine: an evolutionarily ancient but functionally new compound in plants. *Methods Mol. Biol.* 1694 51–59. 10.1007/978-1-4939-7398-9_4 29080154

[B31] Takano A. Kakehi J.-I. Takahashi T. (2012). Thermospermine is not a minor polyamine in the plant kingdom. *Plant Cell Physiol.* 53 606–616. 10.1093/pcp/pcs019 22366038

[B32] Tran M. K. Schultz C. J. Baumann U. (2008). Conserved upstream open reading frames in higher plants. *BMC Genomics* 9:361. 10.1186/1471-2164-9-361 18667093 PMC2527020

[B33] Vera-Sirera F. De Rybel B. Úrbez C. Kouklas E. Pesquera M. Álvarez-Mahecha J. C. (2015). A bHLH-based feedback loop restricts vascular cell proliferation in plants. *Dev. Cell* 35 432–443. 10.1016/j.devcel.2015.10.022 26609958

[B34] von Arnim A. G. Jia Q. Vaughn J. N. (2014). Regulation of plant translation by upstream open reading frames. *Plant Sci.* 214 1–12. 10.1016/j.plantsci.2013.09.006 24268158

[B35] Yamamoto M. Takahashi T. (2017). Thermospermine enhances translation of *SAC51* and *SACL1* in *Arabidopsis*. *Plant Signal. Behav.* 12 e1276685. 10.1080/15592324.2016.1276685 28045577 PMC5289521

[B36] Yoshimoto K. Noutoshi Y. Hayashi K. Shirasu K. Takahashi T. Motose H. (2012). A chemical biology approach reveals an opposite action between thermospermine and auxin in xylem development in *Arabidopsis thaliana*. *Plant Cell Physiol.* 53 635–645. 10.1093/pcp/pcs017 22345435

[B37] Yoshimoto K. Takamura H. Kadota I. Motose H. Takahashi T. (2016). Chemical control of xylem differentiation by thermospermine, xylemin, and auxin. *Sci. Rep.* 6:21487. 10.1038/srep21487 26879262 PMC4754900

