# Supplementary material for: Complexity and Conservation of Thermospermine-Responsive uORFs of SAC51 Family Genes in Angiosperms
Source: Front Plant Sci. 2019 May 1;10:564. doi: 10.3389/fpls.2019.00564 (PMC6504692; doi:10.3389/fpls.2019.00564)
Supplement: Supplementary file 1 [file Table_1.DOCX]

**Table S1.** List of PCR primers used for plasmid construction.

=====================================================================

Primer name Primer sequence Note

------------------------------------------------------------------------------------------------------------------------

SAC51-proFCl 5'-ATCGATTCCCGTGCAATTCTTTA-3' *Arabidopsis thaliana*

SAC51-proRBg 5'-AGATCTAAGTGACCAACGAACA-3'

SAC51-ex2RBg 5'-AGATCTTCCTCTTATACACACTC-3'

SAC51-m1R 5'-GCAATACACAAGCTGTTTTGTGTTT-3'

SAC51-m1F 5'-AAAACAGCTTGTGTATTGCCGTATA-3'

SAC51-m2R 5'-TGTGATCCCAATTTCAAAACTCTTT-3'

SAC51-m2F 5'-TTTTGAAATTGGGATCACAACATTT-3'

SAC51-m3R 5'-ATGGTACACGTAGAAATGTTGTGAT-3'

SAC51-m3F 5'-ACATTTCTACGTGTACCATTGTGAA-3'

SAC51-m4R 5'-TGGCACACCGTAACTTTGAGGAAAT-3'

SAC51-m4F 5'-TCAAAGTTACGGTGTGCCAATCACC-3'

SAC51-m5R 5'-GTTGATAGCACTCAATAACTCTCAC-3'

SAC51-m5F 5'-GTTATTGAGTGCTATCAACCGATGG-3'

SAC51-m6R 5'-CAATTATCCGTCGGTTGATAGCATT-3'

SAC51-m6F 5'-ATCAACCGACGGATAATTGCCAGGT-3'

SAC51-5FSp 5'-ACTAGTCACCTCTTTAGAAATATTTC-3'

SAC51-5RBg 5'-AGATCTTAGCAGTTTAGCTCAAGG-3’

SAC51-5FBal 5'-TGGCCACTTTCATCTCTCTTATCC-3’

SAC51-5RBal 5'-TGGCCAGTTTAGCTCAAGGGAACT-3’

------------------------------------------------------------------------------------------------------------------------

BoSACL1-5F 5'-TCTAGAATTTGCGTTCGCTCCCTC-3' *Brassica oleracea*

BoSACL1-5R 5'-AGATCTCATGCTCTTGCAGTTTAAGCC-3'

BoSACL3-5F 5'-ACTAGTCTTTGCCAATCTTATCTCTC-3'

BoSACL3-5R 5'-GGATCCCATTAGAGAAACCCGCGCAA-3'

GmSACL2-5F 5'-ACTAGTTTCCACATCCACTATCCAC-3' *Glycine max*

GmSACL2-5R 5'-AGATCTcattcaatcagaacaatcacct-3'

GmSACL3-5F 5'-TCTAGAAAAATTGTTCCCAAAAAAACTA-3'

GmSACL3-5R 5'-GGATCCcattatattatctgttcaatct-3'

OsSACL1A-5F 5'-ACTAGTGCAGCGACGAGAAAAAGG-3' *Oryza sativa*

OsSACL1A-5R 5'-AGATCTGGGAAAGCAGGAAATGTT-3'

OsSACL1C-5F 5'-TCTAGACACACACATCCCATGCTACT-3'

OsSACL1C-5R 5'-GGATCCTCTACCTGATCTGATGTAG-3'

OsSACL2-5F 5'-TCTAGAGGGGGCCGATAAGAGAAGC-3'

OsSACL2-5R 5'-GGATCCCATCAAGAAACAACCAGCAA-3'

PtSACL2-5F 5'-TCTAGACATAACCTTCTTCTCGTAC-3' Populus trichocarpa

PtSACL2-5R 5'-GGATCCTTCAGCCTTAACCATCCAA-3'

------------------------------------------------------------------------------------------------------------------------

PBI-Cl 5’-AACGCGCTACAGTCTGACGC-3’

GUS 5’-TCACGGGTTGGGGTTTCTAC-3’

GFP-ATG 5’-GGATCCGGTGAGCAAGGGCGA-3’

GFP-3 5’-GGCGGCCGCTTTACTTGT-3’

=====================================================================

Restriction sites used for cloning are underlined. Nucleotides corresponding to mutated ATG codons are double-underlined.
